# Multiple Attention Mechanism Graph Convolution HAR Model Based on Coordination Theory

**DOI:** 10.3390/s22145259

**Published:** 2022-07-14

**Authors:** Kai Hu, Yiwu Ding, Junlan Jin, Min Xia, Huaming Huang

**Affiliations:** 1 School of Automation, Nanjing University of Information Science and Technology, Nanjing 210044, China; 20191223014@nuist.edu.cn (Y.D.); 20201249090@nuist.edu.cn (J.J.); xiamin@nuist.edu.cn (M.X.); 850028@nuist.edu.cn (H.H.); 2 Jiangsu Collaborative Innovation Center of Atmospheric Environment and Equipment Technology (CICAEET), Nanjing University of Information Science and Technology, Nanjing 210044, China

**Keywords:** human action recognition, graph neural network, attention module

## Abstract

Human action recognition (HAR) is the foundation of human behavior comprehension. It is of great significance and can be used in many real-world applications. From the point of view of human kinematics, the coordination of limbs is an important intrinsic factor of motion and contains a great deal of information. In addition, for different movements, the HAR algorithm provides important, multifaceted attention to each joint. Based on the above analysis, this paper proposes a HAR algorithm, which adopts two attention modules that work together to extract the coordination characteristics in the process of motion, and strengthens the attention of the model to the more important joints in the process of moving. Experimental data shows these two modules can improve the recognition accuracy of the model on the public HAR dataset (NTU-RGB + D, Kinetics-Skeleton).

## 1. Introduction

With the rapid development of artificial intelligence algorithms, motion-recognition technology, which is an important part of artificial intelligence, is being studied for its application in many fields, such as human–computer interaction, video surveillance, film and television production, and other areas [1,2,3]. Many researchers [4,5,6] have invested a great deal of energy in this field and designed many excellent algorithms. Among them, most of the traditional algorithms use manual feature extraction, and these algorithms have made a breakthrough [7]. With the rapid development of machine learning and deep learning, many end-to-end motion recognition algorithms have appeared. These methods do not need to consume a lot of manpower and can achieve high recognition accuracy [8,9].

On the one hand, with deep learning and the rapid development of computer hardware, especially GPU, the performance of action-recognition algorithms is getting better and better. These algorithms can recognize more and more complex actions. Action-recognition algorithms based on deep learning can be roughly divided into the following two categories.

(1) The first category is the motion-recognition algorithm based on traditional CNN, RNN, and LSTM networks, for example, two-stream [10], C3D [11], and LSTM [12], and so on. These algorithms use end-to-end methods to train the model, which can effectively reduce the number of parameters and improve the accuracy of model recognition. Karens et al. [13] designed a two-stream model, which can extract the features of space and time latitude at the same time. They creatively fused the models of the two branches, effectively improving the recognition accuracy of the model. Du et al. [11] applied 3D convolution to action-recognition tasks. The model proposed by them can effectively extract the features of spatial and temporal latitude, and proved that 3 × 3 × 3 convolution is more suitable for action-recognition tasks through experiments. Jeff et al. [12] applied the LSTM model to the action-recognition task and proved through experiments that LSTM is more prominent in the features with time series.

(2) Secondly, with the rise of the graph convolution model, a large number of bone-based motion-recognition models have emerged. These models use human bones as the data of the training model. This type of data is not affected by environmental occlusion, complex background, and optical flow interference, which makes the model more robust. Yan et al. [14] used the graph convolution network in the task of action recognition for the first time. They used the graph convolution model to extract the features in the human skeleton map, and combined with the time convolution to extract the features in the time latitude. Kalpit et al. [15] proposed a bone-partition strategy. They use a partition strategy, which effectively fits the task of local graph convolution. Shi et al. [16] creatively proposed an adaptive graph convolution method based on the spatio–temporal graph convolution, which can adaptively learn bone features and further extract the hidden length, direction, and other features in bones.

On the other hand, coordination is not only the key to improving athletes’ technical ability, but also is an essential part of everyday human physical activities. Coordination refers to the ability of each part of an organism to cooperate with other parts in time and space, and to complete actions in an effective manner. Coordination ability can make movements more accurate and subtle, especially periodic movements. Therefore, athletes attach great importance to the training of coordination ability and regard it as an indispensable and important physical quality to develop in order to more effectively compete and improve. Body coordination also includes three categories: force coordination, movement coordination, and space coordination. First, force coordination refers to the coordination ability of each muscle during tension and contraction. The coordination among the active, antagonistic, and supportive muscles is an important factor in muscle tension and contraction. Therefore, strength coordination training is mainly performed to improve the ability of the nervous system, to get more athletes to participate, to improve the degree of muscle fiber synchronization, to improve the coordination of muscles, and to make athletes exert their maximum potential when exerting strength. Secondly, movement coordination refers to the coordination ability that all humans shows when completing a certain action. Strengthening coordination training can improve human sports performance. Therefore, athletes with good movement coordination ability demonstrate the timeliness and economy of sports technology when they complete technical movements. Finally, spatial coordination refers to the body’s coordination and adaptability with regard to its ability to maintain balance when changing its position. The training of spatial coordination ability is mainly performed to improve people’s adaptability to their three-dimensional sense of space (up and down, left and right, front and back), so as to enhance their spatial awareness or position perception [17]. In terms of coordination in motion theory, we associate coordination features with motion-recognition algorithms. Therefore, this paper proposes a coordinated attention module based on coordination theory.

Through the research and learning of the existing algorithms, the author found the following two problems:

(1). According to the theory of human body-motion balance, the body will produce a coordination feature to maintain balance in the process of moving. Learning about this coordination feature was very helpful for understanding action, but the existing models did not make full use of this feature.

(2). Although the graph convolution neural network was successful in the field of action recognition, the limitation of its adjacency matrix led to the model that can only extract features at the neighbor nodes, and cannot extract features from the global perspective.

To solve the above problems, we improve the Two-Stream Adaptive Graph Convolutional Network (2S-AGCN) algorithm and propose a novel multiple attention mechanism graph convolution action-recognition model based on coordination theory (MA-CT). In this paper, a coordinated attention module (CAM) and an important attention module (IAM) are proposed. The important takeaways from these developments are as follows.

(1). The CAM effectively extracts coordination features generated during motion, and simulate the coordination of human movement through the covariance matrix. This module could effectively improve the accuracy of the basic model.

(2). In addition, the IAM directly started from the feature level, captured the changes of features on nodes, and gave more weight to the more important joints. The module could realize plug and play and effectively improve the accuracy of the basic model.

The structure of this paper is as follows. In the first section, this paper briefly introduces the development of action recognition and the previous methods. Section 2 briefly introduces the graph convolution neural network and the related knowledge of attention mechanism. Section 3 introduces the graph convolution action recognition model based on multiple attention modules, and introduces the details of the two attention modules in detail. In Section 4, experiments are carried out on two large public datasets to verify the effectiveness of the module proposed in this paper, and the model in this paper is compared with the existing model. Section 5 is the summary and prospect of this paper.

## 2. Related Works

### 2.1. Graph Convolution Neural Network

The graph convolution neural network (GCN) [18,19,20,21] summarized the convolution operation from grid image data to graph data with a topological structure. Its main idea was to aggregate the characteristics of its nodes and the characteristics of neighbor nodes, coupled with the natural constraints of the topological graph so that new node characteristics could be generated. The motivation of GCN comes from the combination of convolutional neural networks (CNN) [22,23,24] and topological graphs. With the further development of GCN, graph convolution neural networks could be divided into graph convolution neural networks based on spectral method and graph convolution neural networks based on the spatial method. Kipf et al. proposed a convolution formula combined with a graph Laplacian under the background of spectrum graph theory; however, the spatial-based method was intended to directly convolute the structure of the graph and its neighborhood, and then extract and normalize it according to the manually designed rules. After that, more and more scholars devoted themselves to the task of studying graph convolution neural networks. The fundamental reason is human bone data is topology type data, whereas CNN can only deal with two-dimensional grid data-like images, which is not competent for most tasks in human life. Therefore, in the field of action recognition, more and more people are engaged in the research of graph neural networks because the skeleton data is represented as a topological graph structure rather than a sequence or 2D grid structure.

### 2.2. Study on Action Coordination

**Figure 1:**
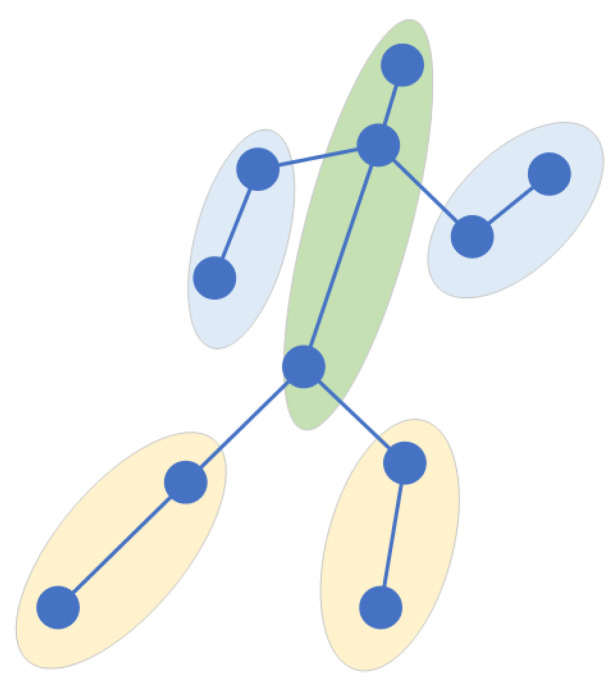
Running: position diagram of arms, legs and body.

Sports cannot be played without the intensively cultivated body coordination of athletes. To improve sports performance, athletes also need to carry out coordination training. Existing algorithms in the field of action recognition do not make full use of the coordination features of the body. Therefore, after consulting many books and papers on basic theories and training methods related to coordination, we chose the skeleton-based action-recognition dataset to deeply study the specific expression of body coordination. Among our findings, we learned that the coordination of the human body requires the sense of space when moving. This sense of space refers to the orientation of each part of the body when moving. Take running as an example. As shown in Figure 1, when a human is running, his hands and legs always swing alternately one after the other, and the arms and legs on the same side must be one after the other. According to the characteristics of the sense of motion space in the coordination of body movement, we studied how to extract the coordination features in the process of movement. To this end, we roughly divide the human body into five areas, including the left arm, the right arm, the left leg, the right leg, and the trunk, which includes the head. The position feature is expressed through appropriate expression, and the position relationship between two pairs is calculated. The coordination feature generated by human motion is calculated through this relationship. In this paper, the local center of gravity theory in physics and the covariance matrix in mathematics are used to express the coordination characteristics of the body.

## 3. Proposed Methods

In recent years, GCN has been used successfully in the field of motion recognition. On the one hand, because human bone data is not affected by interference information such as optical flow and occlusion, the data is purer. On the other hand, the topology of human bone data is a beat set with a graph neural network. The first section investigates the advantages and disadvantages of the existing algorithms in detail. When dealing with the task of human motion recognition based on bone data, these models ignore the coordination features of human action and cannot pay good attention to the more important joints in the process of motion due to the limitation of GCN. On the one hand, the theory of human movement balance [17] describes how the body acts in order to prevent the act of falling and the body’s need to constantly adjust its posture to keep the position of the center of gravity unchanged. In particular, athletes can maintain their balance by swinging their arms and stretching their legs. For ordinary people, everyday actions are also needed to maintain balance, and the cooperation of limbs and trunk is needed to ensure that people will not fall to the ground. Therefore, in the process of completing a certain action, people’s limbs have roughly fixed movement tracks. For example, in the action of running, when the left foot moves forward, the right arm must swing back to keep the position of the body’s center of gravity unchanged; otherwise, there will be a risk of falling. On the other hand, the importance of different joints in different human actions is different, and these more important joints often number more than one. The existing models fail to pay good attention to the extraction of this part of the features. In addition, due to the fixity of the physical connection of the human body, the GCN is often fixed when extracting features and fails to pay better attention to the mutual features of several more important joints from a global perspective. These joints are often not connected in most actions. For example, in the action of clapping hands, from the perspective of the human skeleton map, the nodes of both hands are not directly connected and are far apart. However, both hands are an important part of the action of clapping hands, and the changes of various characteristics also focus on both hands. To solve the above problems, we propose two attention modules, namely the coordination attention module and the importance attention module, to solve the above two problems.

### 3.1. Multiple Attention Mechanism Graph Convolution Action-Recognition Model Based on Action Coordination Theory

Based on the 2S-AGCN algorithm, we propose a multiple attention mechanism graph convolution action-recognition model based on action coordination theory (MA-CT). The model solves some problems and helps the model to better identify the categories of human actions. On the one hand, the coordinated attention module (CAM) is mainly used to extract the coordination features generated in the process of human movement, and use this coordination feature to further strengthen the input of the model. On the other hand, the importance of attention module (IAM) aims to solve the problem that the model is limited by the graph convolution neural network, which makes the model unable to observe the more important joints in the movement process through the global field of vision. This section mainly introduces the original adaptive graph convolution model structure, the multiple attention mechanism graph convolution action-recognition model structure based on action coordination theory, and the structure of two attention modules.

#### 3.1.1. Adaptive Graph Convolution Module

**Figure 2:**
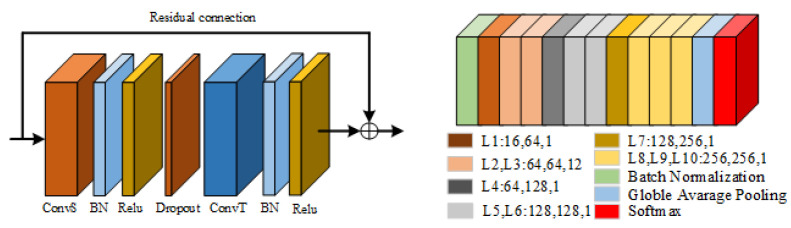
Original adaptive graph convolution module (**left**) and adaptive graph convolution model (**right**) [7].

We take 2S-AGCN as the basic model that was introduced in detail in our other paper [5]. This article will briefly introduce the prominent contents. As shown in Figure 2, an adaptive graph convolution network is used to stack the above adaptive graph convolution modules. There are nine modules in total. The numbers of output channels of each module are 64, 64, 64, 128, 256, 256, and 256. Before the beginning of the network, add a BN layer to standardize the input data, add global average pooling after the ninth module, and finally input the results into the softmax layer to obtain the predicted result. The calculation formula of adaptive graph convolution is shown in Equation (1),

(1) $$f_{out}=\sum_{k}^{K_{v}}W_{k}f_{in}\left(A_{k}+B_{k}+C_{k}\right),$$

where $K_{v}$ is the kernel size of the spatial dimension and set to 3, $W_{k}$ is the weight matrix. $A_{k}$, $B_{k}$, and $C_{k}$ is three kinds of the adjacency matrix.

Here we will focus on the calculation process of $C_{k}$. $C_{k}$ can learn a unique graph for each sample. To determine whether there is a connection between two adjacent nodes and how strong the connection is, we use the normalized Gaussian embedding function to calculate the similarity of the two nodes, as shown in Equation (2):

(2) $$f\left(v_{i},v_{j}\right)=\frac{e^{\theta }{\left(v_{i}\right)}^{T}\Phi \left(v_{j}\right)}{\sum_{j=1}^{N}e^{\theta }{\left(v_{i}\right)}^{T}\Phi \left(v_{j}\right)}.$$

#### 3.1.2. Multiple Attention Mechanism Graph Convolution Action-Recognition Model Based on Action Coordination Theory

**Figure 3:**
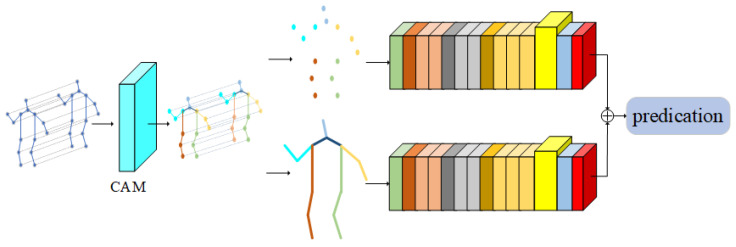
Multiple attention mechanism convolution action-recognition model based on action coordination theory (MA-CT).

In this section, aiming at the existing models cannot effectively use the coordination characteristics of the body in the process of human movement, and due to the limitations of the graph convolution network, it is impossible to obtain the importance of joints from the global field of vision; therefore, a multiple attention mechanism graph convolution action-recognition model based on motion coordination theory is proposed. The overall framework of the model is shown in Figure 3. The light blue square in Figure 3 represents the CAM proposed in this paper, and the highlighted part in yellow represents the new adaptive graph convolution network after inserting IAM.

The multi-attention mechanism graph convolution action-recognition model based on the action coordination theory proposed in this paper is an end-to-end training model. The overall framework can be roughly divided into three parts: coordination attention module, dual flow adaptive graph convolution model, and importance attention module. The model is based on the 2S-AGCN algorithm. After inputting the action sequence, the coordination attention module is used to preprocess the original data, mine the coordination characteristics of human action, and obtain a group of action sequences with coordination characteristics, which effectively integrates the concept of body coordination in human motion theory into the deep learning model. Then, according to the idea of the dual flow adaptive graph convolution model, the new action sequence is decomposed into two parts; one is a node feature, and the other is bone feature. Among them, the node characteristics include the coordinates on the node, confidence, and so on. Bone length, orientation, and other features are included. The two sets of data are used as the input of two identical adaptive graph convolutions for feature extraction. After the ninth layer of the adaptive graph convolution model, the features are input into the importance attention module, which can pay attention to the more important joints in the movement process, which effectively solves the deficiency that the existing models cannot obtain the important joints through the global field of view. Finally, through the softmax layer, two classification results are obtained, respectively. Finally, the two classification results are fused to obtain the final classification result of this model.

### 3.2. Coordination Attention Module

**Figure 4:**
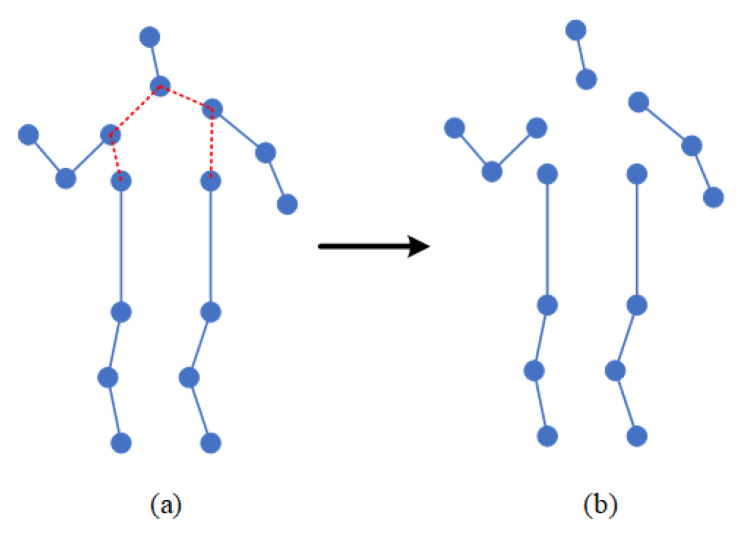
Partition strategy of human skeleton map. (**a**) shows the unprocessed human skeleton diagram, in which the red connecting part represents the divided connecting line, and (**b**) shows the human skeleton diagram after being divided into five partitions.

In the process of movement, people are always maintaining balance, which requires the cooperation of limbs and the trunk. Therefore, in the process of movement, the position and trajectory of each body part are roughly fixed. Inspired by this idea, the coordination of human motion is introduced into the action-recognition model. Therefore, this paper proposes a coordinated attention module, which is a computing unit, which is composed of the bone-partition strategy, matrix calculation, covariance matrix, and so on. The bone-partition strategy of the coordinated attention module proposed in this paper is shown in Figure 4. According to the structure of the human body, the human bone map is divided into five partitions, including the head, left arm, right arm, left leg, and right leg, and five subgraphs are obtained.

Then the model calculates the center-of-gravity point of each region. Mathematically and physically, it is stipulated that the center of gravity is closely related to the balance of the object, the motion of the object, and the internal force distribution of the constituent object. The author considers that to reduce the calculation amount of the model, the module uses the center of gravity of each region to calculate the coordination, which will be much less than the calculation amount of directly using joints, and can effectively avoid the problem of inconsistent nodes of each part. According to Equation (3), the center of gravity points on the five sub-graphs are calculated, respectively, and the center-of-gravity coordinates of each part are calculated to represent the general position of the area. The general motion trajectory of each area can be obtained by tracking the motion trajectory of the center of gravity. Let the center-of-gravity matrix be $\left(w_{1},w_{2},w_{3},w_{4},w_{5}\right)$. *n* in Equation (3) represents the number of nodes, and $x_{n}$ represents the value of the abscissa of the *n*th node. Here, to simplify the expression, only the calculation formula of abscissa is shown, and the calculation of the other two coordinates is consistent with Equation (3):

(3) $$w=\frac{\left(x_{1}+x_{2}+\ldots +x_{n}\right)}{n},n=1,2,\cdots ,n.$$

**Figure 5:**
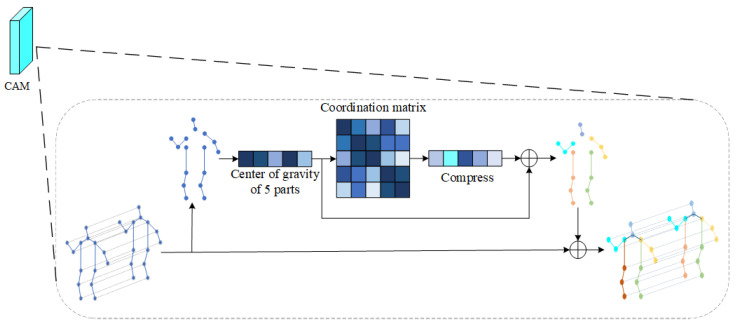
Coordination attention module.

As shown in Figure 5, according to Equation (3), the center-of-gravity points in five zones can be obtained. Then calculate the body coordination matrix. Covariance is widely used in statistics and machine learning. Statistically, covariance is generally used to describe the similarity between two variables, and variance is a special case of covariance. The author believes that the covariance matrix can be used to calculate the similarity between various regions, and the similarity between two barycenters can be used to express the coordination of the body. The module introduces the covariance matrix into the action-recognition module to calculate the coordination relationship between two regions. The following will introduce the specific calculation methods of covariance and variance and rewrite the calculation of the covariance matrix according to the characteristics of the data used in this paper to make it more consistent with said data. The standard variance and covariance are calculated as shown in Equations (4) and (5).

(4) $$s^{2}=\frac{\sum_{i-1}^{n}{\left(X_{i}-\bar{X}\right)}^{2}}{n-1},i=1,2,\cdots ,n$$

(5) $$cov\left(X,Y\right)=\frac{\sum_{i-1}^{n}\left(X_{i}-\bar{X}\right)\left(Y_{i}-\bar{Y}\right)}{n-1},i=1,2,\cdots ,n$$

Here, *s* represents variance, *X* and *Y* represent two groups of random variables, cov(X,Y) represents the covariance of variables *X* and *Y*, *i* represents the *i*th variable in *X* or *Y*, and *n* represents the number of samples. According to the characteristics of the data in this paper, combined with Equations (4) and (5), we rewrite the covariance matrix into a form suitable for application in this paper. Here, *n* is set to 5, samples *X* and *Y* are set to the same sample, and the values are consistent, which is the center-of-gravity matrix. Let $X_{i}=Y_{j}=\left(w_{1},w_{2},w_{3},w_{4},w_{5}\right),i=j=1,2,3,4,5$. Rewrite Equation (5) to obtain the calculation formula of coordination matrix used in this module, as shown in Equation (6):

(6) $$cov\left(X,Y\right)=\frac{\sum_{i-1}^{5}\left(X_{i}-\bar{X}\right)\left(Y_{i}-\bar{Y}\right)}{4},i=1,2,3,4,5.$$

According to Equation (6) and the center-of-gravity matrix, the coordination matrix related to each other can be calculated. The matrix form is shown in Equation (7). Similarly, the coordination matrix of the remaining two coordinates can be calculated by using Equation (6).

(7) $$\begin{array}{c} cov(X,Y)=\\ \left[\begin{array}{ccccc} cov(w_{1},w_{1}) & cov(w_{1},w_{2}) & cov(w_{1},w_{3}) & cov(w_{1},w_{4}) & cov(w_{1},w_{5})\\ cov(w_{2},w_{1}) & cov(w_{2},w_{2}) & cov(w_{2},w_{3}) & cov(w_{2},w_{4}) & cov(w_{2},w_{5})\\ cov(w_{3},w_{1}) & cov(w_{3},w_{2}) & cov(w_{3},w_{3}) & cov(w_{3},w_{4}) & cov(w_{3},w_{5})\\ cov(w_{4},w_{1}) & cov(w_{4},w_{2}) & cov(w_{4},w_{3}) & cov(w_{4},w_{4}) & cov(w_{4},w_{5})\\ cov(w_{5},w_{1}) & cov(w_{5},w_{2}) & cov(w_{5},w_{3}) & cov(w_{5},w_{4}) & cov(w_{5},w_{5}) \end{array}\right] \end{array}$$

According to Equation (7), three groups of coordination matrices can be obtained. These three groups of coordination matrices are expressed as $w_{x}$, $w_{y}$, and $w_{z}$, respectively. These three groups of matrices can be used to represent the coordination characteristics of the body. Compress $w_{x}$, $w_{y}$, and $w_{z}$ to the same size as the dimension of the center-of-gravity matrix. The compression method here is in the form of column-by-column addition, as shown in Equation (8). Take the first column as an example to illustrate the compression method.

(8) $$X_{i}=cov\left(w_{1},w_{1}\right)+cov\left(w_{2},w_{1}\right)+cov\left(w_{3},w_{1}\right)+cov\left(w_{4},w_{1}\right)+cov\left(w_{5},w_{1}\right)$$

Add the barycentric matrix and the compressed coordination matrix to obtain the barycentric matrix $\left({\dot{w}}_{1},{\dot{w}}_{2},{\dot{w}}_{3},{\dot{w}}_{4},{\dot{w}}_{5}\right)$ with coordination characteristics. Here, we consider the operation of matrix multiplication, but the coordinate values of most points are less than 1. If the matrix is multiplied, it will be smaller, and even lead to the loss of features. Finally, the center-of-gravity matrix is added to each node according to the region, so that a set of bone data with coordination characteristics can be obtained.

### 3.3. Importance Attention Module

**Figure 6:**
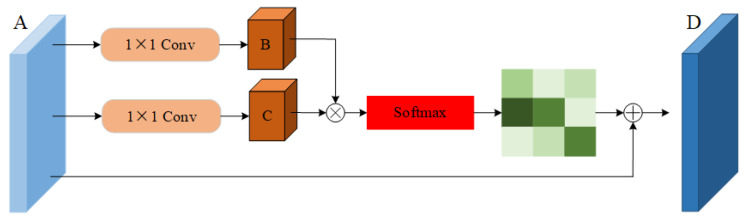
Importance attention module (IAM).

The graph volume model processes the data of the topology structure, which is in good agreement with the action-recognition task based on the human skeleton graph. At present, many models have achieved very good results. However, these models still have some shortcomings in the global field of view. Due to the limitation of human body topology, it is difficult for the graph volume model to learn the relationship between various end nodes, which is often an important part of the action. In addition, the deep graph convolution model easily leads to the phenomenon of excessive smoothing of features [25,26,27], so it is not suitable to use the deep model [28,29,30]. Inspired by the dual attention network (DA-net) [31,32], an attention module is proposed. DA-net can capture the global feature dependencies in both spatial and channel dimensions. The model uses the location attention module to learn the spatial interdependence of features and designs the channel attention module to simulate the interdependence between channels. Inspired by this idea, the location attention mechanism is embedded into the adaptive graph convolution model to obtain the important features of nodes in the feature graph and transfer them to the original feature graph. This paper proposes an important attention module. When extracting features, the module operates directly on the feature map, which can effectively overcome the limitations of the graph convolution neural network. The important attention module proposed in this paper is shown in Figure 6. The input of this module is the feature map obtained after spatial map convolution sampling and time convolution sampling, and the output is the feature map with attention characteristics.

Because the number of channels in the ninth layer of the adaptive graph convolution model has reached 256, the value is too large, and the calculation size in the process of parameter transmission is large. To reduce the computational burden, use the convolution of 11 reduces the dimension of the feature channel, which effectively reduces the amount of calculation. First, the characteristic diagram in Figure 6 is divided into three branches, $A\in R^{(N\times M)\times C\times T\times V}$, where (N×M) represents the product of the batch size and the number of characters, *C* indicates the number of channels, *T* indicates the number of action frames, and *V* indicates the number of nodes. Then, *A* is sent into two convolution layers of 11 to obtain two new feature maps *B* and *C*, $\left\{B,C\right\}\in R^{(N\times M)\times C\times T\times V}$. Then the characteristic figure *B* and *C* are reconstituted into $R^{C\times D}$, D=(N×M)×T×V, where *D* represents the number of feature points on each channel. Then the transposition of *B* and *C* is matrix-multiplied, and the position attention feature map *S* is calculated by the softmax layer, $S\in R^{D\times D}$. The calculation formula of the attention characteristic map is shown in Equation (9). where $s_{ji}$ represents the influence of the *i*th position on the *j*th position:

(9) $$S_{ji}=\frac{\exp(B_{i}\cdot C_{j})}{\sum_{i=1}^{N}\exp(B_{i}\cdot C_{j})}.$$

At the same time, the feature map *S* is reorganized and multiplied by a scale coefficient α, which is added to the feature map a to obtain the final output *D*, $D\in R^{(N\times M)\times C\times T\times V}$. The initial value of α is set to 0 and can gradually learn greater weight. The feature *D* of each position is the weighted sum of all position features and the original features. Therefore, it has a global vision and can selectively aggregate context information according to the spatial attention map:

(10) $$E_{j}=\alpha \sum_{i=1}^{N}s_{ji}+A_{j}.$$

Here, the initial value of α is set to 0, and the corresponding weight can be gradually obtained through training.

**Figure 7:**
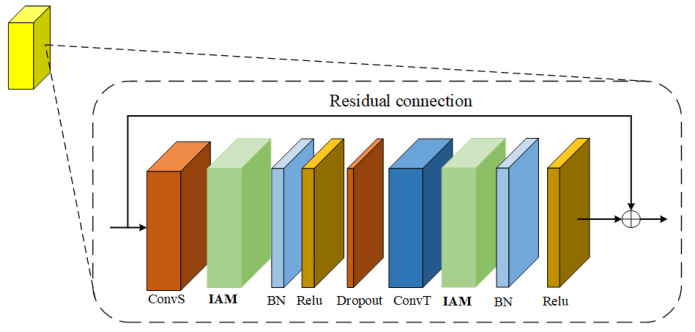
Adaptive graph convolution model with importance attention module.

The importance attention module proposed in this paper can realize plug and play. The author puts it after the space graph convolution and time convolution in the adaptive graph convolution model. As shown in Figure 7, the more important joints in the process of human motion are extracted from the space dimension and time dimension respectively.

**Figure 8:**
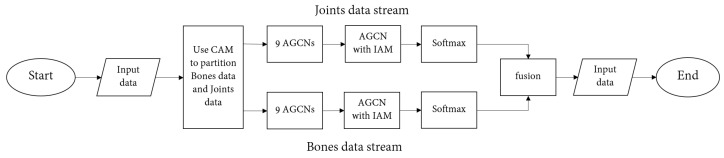
Flowchart of the methodology.

In order to better explain the algorithm proposed in this paper, we simply provide an algorithm flow chart, as shown in Figure 8.

## 4. Experimental Results and Analysis

This section verifies the effectiveness of the coordination attention module and importance attention module proposed in this paper through experiments. To facilitate the comparison with the initial model 2S-AGCN, experimental verification is carried out on two large datasets: Kinetics-Skeleton and NTU-RGB + D. When verifying the coordination attention module, this section compares each branch of the two-stream network and then compares the results of the two-stream fusion. When verifying the importance attention module, because this paper inserts the importance attention module in two positions, to verify its effectiveness this section verifies the effectiveness of the importance attention module in space and time dimensions respectively. Then the two modules are fused to verify the effectiveness of the spatio–temporal importance attention module. Finally, the graph convolution motion recognition model based on multiple attention modules proposed in this paper is compared with the model on the same dataset to verify its effectiveness.

### 4.1. Datasets and Experimental Details

#### 4.1.1. NTU-RGB + D

NTU-RGB + D [33] is one of the largest datasets in the human action-recognition task and contains 56,000 action clips in 60 action classes. Each action is taken with three cameras. The dataset gives the position information of nodes in each frame. There are 25 nodes in each frame. The author of this dataset two proposed benchmarks—cross-subject (X-Sub) and cross-view (X-View)—in his paper [33]. The former divides the training set and the test set according to the subject, and the latter divides the training set and the test set according to the camera number.

#### 4.1.2. Kinetics-Skeleton

Kinetics [34] is another of the largest human action datasets, and contains 400 action categories. These video clips are taken from YouTube. We use the OpenPose toolbox to extract bone data from these videos, and extract bone data with 18 key points from the video sequence. In this paper, we use their released data (Kinetics-Skeleton) to evaluate the model in this paper. This dataset can be divided into a training set and a verification set. The training set has 240,000 segments, and the verification set includes 20,000 segments.

#### 4.1.3. Training Details

All the experiments in this paper were completed under the same equipment. The hardware condition of the device was the ninth-generation Intel CPU, 64 g RAM and two 2080 Ti GPUs. The software condition was based on the Pytoch framework. The optimization algorithm was the stochastic gradient descent (SGD). Its momentum was set to 0.9. The cross-entropy loss function was used, and the initial learning rate was set to 0.1. For the NTU-RGBD and Kinetics-Skeleton datasets, due to the limitations of the experimental conditions in this paper, we set the batch size of the model to 16. The learning rate is set as 0.1 and is divided by 10 at the 30th epoch and the 40th epoch. The training process is ended at the 50th epoch [16]. For the Kinetics-Skeleton dataset, the size of the input tensor of Kinetics is set the same as [16], which contains 150 frames with two bodies in each frame. We perform the same data-augmentation methods as in [16]. In detail, we randomly choose 300 frames from the input skeleton sequence and slightly disturb the joint coordinates with randomly chosen rotations and translations. The learning rate is also set as 0.1 and is divided by 10 at the 45th epoch and 55th epoch. The training process is ended at the 65th epoch [16]. To enhance the accuracy of the experimental results, we did 10 experiments and took the average value as the final experimental results.

### 4.2. Ablation Experiment

#### 4.2.1. Effectiveness Analysis of Coordination Attention Module

To verify the effectiveness of the coordination attention module (CAM) proposed in this paper, this section uses two large datasets—NTU-RGB + D and Kinetics-Skeleton—and compares the effectiveness of the coordination module by controlling variables. Under the same hardware conditions and the same parameter settings, the results shown in Table 1 are obtained, in which “J-Stream” and “B-Stream” respectively represent the joint stream and bone stream of the 2S-AGCN, and “CAM” represents the abbreviation of the coordinated attention module proposed in this paper. On the CV index of the NTU-RGB + D dataset, the accuracy of “J-Stream” of the initial 2S-AGCN is 93.1%, the accuracy of “B-Stream” is 93.3%, and the accuracy after two-stream fusion is 95.1%. In terms of CS index, the accuracy of “J-Stream” in the experimental environment is 86.3%, the accuracy of “B-Stream” is 86.7%, and the accuracy after two-stream fusion is 88.5%. In the Kinetics-Skeleton dataset, the accuracy of “J-Stream” in the experimental environment is 34.0%, that of “B-Stream” is 34.3%, and that of 2S-AGCN is 36.1%.

Under the same test conditions, the CAM proposed in this paper is inserted into the adaptive graph convolution model. In the CV index of the NTU-RGB + D dataset, the accuracy of “CAM + J-Stream” is 94%, which is 0.9% higher than the original accuracy. The accuracy of “CAM + B-Stream” is 93.5%, which is 0.2% higher than the original accuracy. The accuracy of two-stream fusion is 95.3%, which is 0.2% higher than the original accuracy. In terms of CS index, the accuracy of “CAM + J-Stream” is 86.9%, which is 0.6% higher than the original accuracy. The accuracy of “CAM + B-Stream” is 87.5%, which is 0.8% higher than the original accuracy. The accuracy of two-stream fusion is 88.8%, which is 0.3% higher than the original accuracy. In the Kinetics-Skeleton dataset, the accuracy of “CAM + J-Stream” is 35.4%, which is 1.4% higher than the original accuracy. The accuracy of “CAM + B-Stream” is 34.5%, which is 0.2% higher than the original accuracy. The accuracy of two-stream fusion is 36.5%, which is 0.4% higher than the original accuracy.

It can be seen from Table 1 that the performance of the adaptive graph convolution model combined with the coordinated attention module has improved in the two datasets. The module calculates the barycenter positions of the five partitions of the body, then calculates the relationship between the five locations by using the covariance matrix, and adds it to the features as a coordination matrix, which enriches the representation of the features. From the experimental results, this module can improve the accuracy of the model. After the two-stream fusion, the effect is better than the model without the coordination feature.

**Figure 9:**
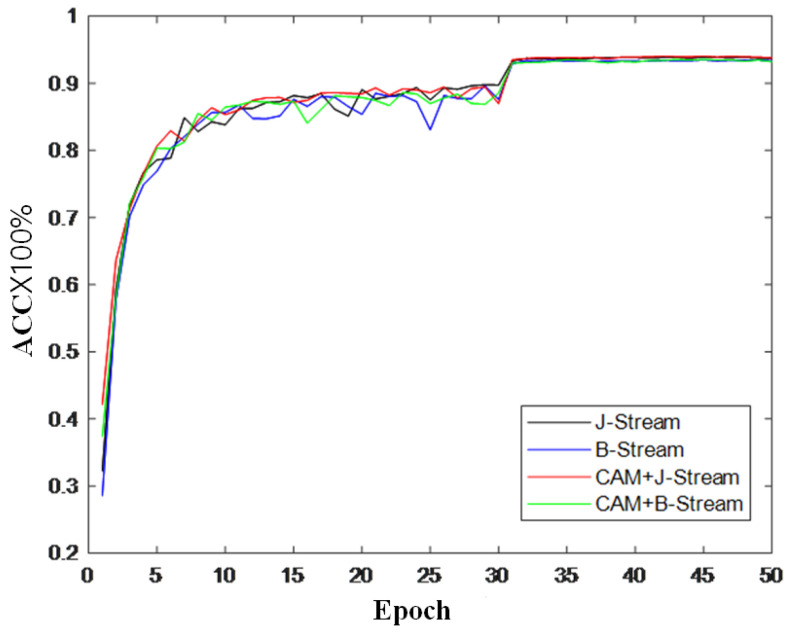
Accuracy change curve on CV index of NTU-RGB + D.

**Figure 10:**
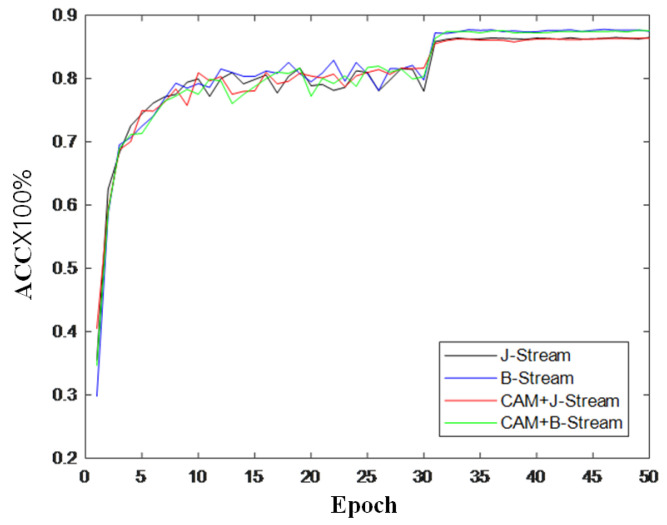
Accuracy change curve on CS index of NTU-RGB + D.

**Figure 11:**
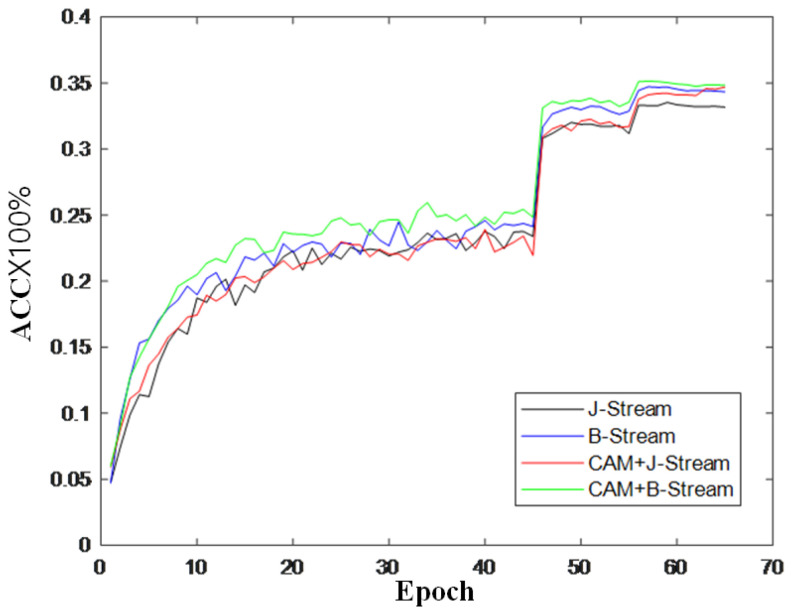
Accuracy change curve of Kinetics-Skeleton.

To further verify the effectiveness of the module, this section also records the changes in accuracy during model training and draws a curve to compare the changes in inaccuracy, as shown in Figure 9, Figure 10 and Figure 11. It can be seen from the accuracy curve that the coordinated attention module proposed in this paper is better able to help the model understand the action semantics. In the two datasets, the initial accuracy of the model is higher than that of the original two-stream adaptive graph convolution model, and the oscillation amplitude of the accuracy is also small in the training process. When the final model tends to converge, the accuracy is also improved to a certain extent, which shows that the coordination attention module can effectively extract the coordination features of human bones, and provide help for the discrimination of action semantics.

#### 4.2.2. Effectiveness Analysis of Importance Attention Module

Aiming at the shortcomings of the existing models, this paper proposes an importance attention module (IAM). The module can observe the changes in joints from a global perspective and can calculate the dependencies between non-adjacent nodes from the topology. This module can realize plug and play. Because the adaptive graph convolution module extracts the features of the data in both space and time dimensions at the same time, this paper places the importance module after the spatial graph convolution layer and time convolution layer respectively. This section will verify and analyze the effectiveness of the module on two large public datasets. All data in Table 2 are completed under the same parameter settings and hardware conditions. “IAM-S” in the table indicates that the importance attention module is placed after spatial map convolution, “IAM-T” indicates that the importance attention module is placed after time convolution, and “IAM-ST” indicates that the importance attention module is placed at both locations. To facilitate the comparison with the 2S-AGCN, this section compares the accuracy of the importance attention module with the “J-Stream” and “B-Stream” of the initial model and verifies its effectiveness one by one. Then, the results of the two streams are fused to obtain the final classification result. From the experimental results in Table 2, it can be concluded that the IAM will improve the accuracy of the model after the spatial map convolution or time convolution. When the two positions are placed at the same time, the accuracy of the model will be further improved, which shows that the important attention module can effectively observe the joints that are more important for motion understanding from the perspective of global vision. In the CV index of the NTU-RGB + D dataset, after adding two groups of IAMs, the accuracy of the model is 95.7%, which is 0.6% higher than the initial 2S-AGCN. In the CS index, the accuracy of the model is improved by 0.4% compared with the initial model. In the Kinetics-Skeleton dataset, the accuracy of the model is improved by 0.9% compared with the initial model. The results in Table 2 illustrate the effectiveness of the importance attention module proposed in this paper.

### 4.3. Comparison with Other Methods

This paper proposes a convolution action recognition model of multiple attention mechanism graphs based on action coordination theory. The experiments in Section 4.3 confirm the effectiveness of the two attention modules proposed in this paper. This section compares the MA-CT with some existing algorithms in the same datasets. The results of these two comparisons are shown in Table 3 and Table 4, in these tables, bolded data is best. The methods used for comparison include the handcrafted feature-based method [35], RNN-based methods [36,37,38], CNN-based methods [39,40], and GCN-based methods [5,14,16,41,42,43,44,45,46,47]. The accuracy of MA-CT in CV index on NTU-RGB + D is 95.9%, and the accuracy in the CS index is 89.7%. Compared with the original 2S-AGCN, it is improved by 0.8% and 1.2%, respectively. In the Kinetics-Skeleton dataset, the accuracy of MA-CT reaches 37.3%, which is 1.2% higher than that of the original model. At the same time, compared with the model proposed in Section 3 it is improved by 0.2%. As can be seen from Table 3, in terms of the CV index, the model proposed in this paper is still inferior to the more advanced MV-IGNet. However, in terms of the CS index, the accuracy of the model proposed in this paper is 0.3% higher than that of MV-IGNet. It can be seen from Table 3 that the model proposed in this paper has improved upon the initial model, indicating that the coordination attention module and importance attention module can improve the accuracy of model recognition to a certain extent. In the Kinetics-Skeleton dataset, the accuracy of the proposed model in top-1 is 37.3%, which is 1.2% higher than the original 2S-AGCN. The accuracy of this model in top-1 is still not as good as 2S-AAGCN and 4S-AAGCN, but the accuracy of top-5 is 1% and 0.4% higher.

## 5. Discussion

With the rapid development of artificial intelligence and its application in various fields, HAR has become an important area of development through deep learning to identify human movement. There is still room for further improvement in the accuracy of current HAR algorithms before its best engineering applications can be achieved.

In the development of existing HAR algorithms, people are always accustomed to introducing newly developed deep learning algorithms into HAR algorithms, which has played a role in improving the accuracy. Compared with traditional machine learning, deep learning essentially uses complex networks for automatic learning data features. In order to achieve better learning of such features, the network of deep learning becomes more and more complex, which requires more expensive hardware, and the requirement is contradictory to engineering application. Therefore, if the network structure remains unchanged (the requirements for hardware also remain unchanged), artificial emphasis on some prior knowledge and enhancement of some features will enable the network to quickly grasp these important features, and improve the accuracy to become a better choice.

Based on the coordination theory in sports kinematics, and by combining the digital robot control theory and the attention mechanism, this study has some innovations in feature enhancement and model structure. For feature extraction, this study uses a two-channel scheme to extract joint and bones features, which are divided into two data streams for analysis. In the aspect of feature enhancement, the coordination attention module and the importance attention module are designed and used to focus on the correlation of upper and lower frames action coordination, and finally achieve the fusion output. This study improves the accuracy, which proves that the idea of HAR combined with the coordination theory is correct.

In addition, we also recognize that because the learning data and validation data of this algorithm come from generally accepted standard datasets, and most of these standard datasets are stable movements of healthy people, this is obviously a positive sample for whole data, and uncoordinated actions should also be the content of learning and analysis, which is one of the defects of this study. Of course, it is easy to imagine that if human movements were inconsistent and the center of gravity was unstable, the predictable results are falls, so this algorithm should be used to predict the action of human falls.

## 6. Conclusions

In this work, we propose a multiple attention mechanism graph convolution action recognition model based on coordination theory (MA-CT). It parameterizes the graph structure of the skeleton data and embeds it into the network to be jointly learned and updated with the model. This data-driven approach increases the flexibility of the graph convolutional network and is more suitable for the action recognition task. Furthermore, the existing methods do not make full use of the coordination features of human motion, and because of the existence of an adjacency matrix, the model cannot extract features from the global perspective. In this work, we propose a coordination attention module (CAM) and importance attention module (IAM). In this paper, experiments are carried out on two large public datasets. For the two indicators of NTU-RGB + D, the CAM improves the accuracy of the model by 0.2% and 0.3%, and the IAM improves the accuracy of the model by 0.6% and 0.4%. In the Kinetics dataset, the CAM improves the accuracy of the model by 0.4%, and the IAM improves the accuracy of the model by 0.9%. They are used to solve the problems of insufficient feature extraction and the capturing of key joints. The final model has achieved good results in NTU-RGB + D and Kinetics.

## Figures and Tables

**Table 1:** Effectiveness analysis of coordination attention module on NTU-RGB + D and Kinetics-Skeleton datasets.

Methods	NTU-RGB + D		Kinetics-Skeleton (%)
	CV (%)	CS (%)	
J-Stream	93.1	86.3	34.0
B-Stream	93.3	86.7	34.3
2s-AGCN	95.1	88.5	36.1
CAM + J-Stream	94.0	86.9	35.4
CAM + B-Stream	93.5	87.5	34.5
CAM + 2s-AGCN	95.3	88.8	36.5

**Table 2:** Effectiveness analysis of importance attention module in NTU-RGB + D and Kinetics-Skeleton.

Methods	NTU-RGB + D		Kinetics-Skeleton (%)
	CV (%)	CS (%)	
J-Stream	93.1	86.3	34.0
B-Stream	93.3	86.7	34.3
IAM-S + J-Stream	93.9	86.9	34.9
IAM-S + B-Stream	93.5	86.5	34.5
IAM-T + J-Stream	94.4	87.1	35.0
IAM-T + B-Stream	94.1	86.7	34.5
(IAM-ST) + J-Stream	94.6	86.9	34.8
(IAM-ST) + B-Stream	94.3	86.6	34.6
2s-AGCN	95.1	88.5	36.1
IAM-S + 2s-AGCN	95.2	88.6	36.3
IAM-T + 2s-AGCN	95.5	88.7	36.4
(IAM-ST) + 2s-AGCN	95.7	88.9	37.0

**Table 3:** Comparison of accuracy between ours model and other models on NTU-RGB + D dataset.

Methods	CV (%)	CS (%)
Deep LSTM [36]	67.3	60.7
Temporal ConvNet [39]	83.1	74.3
VA-LSTM [37]	87.6	79.4
Two-stream CNN [40]	89.3	83.2
GCA-LSTM [41]	82.8	74.4
ARRN-LATM [38]	89.6	81.8
MANs [42]	93.22	83.01
ST-GCN [14]	88.3	81.5
DPRL + GCNN [43]	89.8	83.5
2S-AGCN [16]	95.1	88.5
RA-GCN [44]	93.6	87.3
MV-IGNet [45]	96.3	89.2
MST-AGCN [5]	95.5	89.5
**MA-CT (ours)**	**95.9**	**89.7**

**Table 4:** Comparison of accuracy between ours model and other models on Kinetics-Skeleton dataset.

Methods	CV (%)	CS (%)
Feature Encoding [35]	14.9	25.8
Deep LSTM [36]	16.4	35.3
Temporal ConvNet [39]	20.3	40.0
ST-GCN [14]	30.7	52.8
2S-AGCN [16]	36.1	58.7
GCN-NAS [46]	37.1	60.0
1s-AAGCN [47]	36.0	58.4
2s-AAGCN [47]	37.4	60.4
4s-AAGCN [47]	37.8	61.0
MST-AGCN [5]	37.1	61.0
**MA-CT (ours)**	**37.3**	**61.4**

## Data Availability

The data and code used to support the findings of this study are available from the corresponding author upon request (001600@nuist.edu.cn).
